# Comparative Assessment of the qSOFA, SII, dNLR, and OISS Infection Severity Scores in Diabetic Versus Non-Diabetic Patients with Odontogenic Infections

**DOI:** 10.3390/biomedicines12122712

**Published:** 2024-11-27

**Authors:** Otilia Cornelia Bolos, Bogdan-Valeriu Sorca, Laura-Cristina Rusu, Gianina Tapalaga

**Affiliations:** 1 Department of Dental Aesthetics, Faculty of Dental Medicine, “Victor Babes” University of Medicine and Pharmacy Timisoara, Revolutiei Boulevard 9, 300041 Timisoara, Romania; bolos.otilia@umft.ro; 2 Department of Oral Pathology, Multidisciplinary Center for Research, Evaluation, Diagnosis, and Therapies in Oral Medicine, Faculty of Dental Medicine, “Victor Babes” University of Medicine and Pharmacy Timisoara, Revolutiei Boulevard 9, 300041 Timisoara, Romania; laura.rusu@umft.ro; 3 Department of Odontotherapy and Endodontics, Faculty of Dental Medicine, “Victor Babes” University of Medicine and Pharmacy Timisoara, Eftimie Murgu Square 2, 300041 Timisoara, Romania; tapalaga.gianina@umft.ro

**Keywords:** odontogenic infections, diabetes mellitus, inflammatory scores, qSOFA, SII, dNLR, OISS

## Abstract

**Background and Objectives:** Odontogenic infections (OIs) can progress rapidly and may lead to severe systemic complications, especially in patients with underlying conditions like diabetes mellitus (DM). This study aims to evaluate the predictive value of inflammatory scores—quick Sequential Organ Failure Assessment (qSOFA), Systemic Immune–Inflammation Index (SII), derived Neutrophil-to-Lymphocyte Ratio (dNLR), and Odontogenic Infection Severity Score (OISS)—in assessing the severity of OIs in diabetic versus non-diabetic patients. **Materials and Methods:** A case–control study was conducted on 123 patients diagnosed with OIs. Patients were divided into two groups: patients with diabetes (*n* = 42) and patients who were non-diabetic (*n* = 81). Inflammatory scores were calculated at admission and correlated with clinical outcomes. Statistical analyses included *t*-tests, chi-square tests, and multivariate logistic regression. **Results:** The patients with diabetes exhibited significantly higher OISS scores (mean 6.5 ± 2.8) compared to the patients who were non-diabetic (mean 4.8 ± 2.1, *p* < 0.001). The inflammatory markers qSOFA, SII, and dNLR were significantly elevated in the diabetic group (all *p* < 0.01). The SII demonstrated the highest predictive accuracy for severe OIs in patients with diabetes, with an area under the curve (AUC) of 0.88 (95% CI: 0.80–0.95). Diabetes mellitus was an independent predictor of severe OIs (OR: 3.2, 95% CI: 1.5–6.8, *p* = 0.003). **Conclusions:** Inflammatory scores, particularly SII, are effective in predicting the severity of odontogenic infections in patients with diabetes. Incorporating these scores into clinical practice may enhance the early identification of high-risk patients and improve management strategies.

## 1. Introduction

Odontogenic infections (OIs) are infections originating from dental tissues or their supporting structures and can lead to significant morbidity if not promptly diagnosed and managed [1,2]. The progression from a localized dental infection to a severe systemic condition can be swift, often resulting in complications such as deep neck space infections, sepsis, and airway obstruction [3]. Understanding the factors that contribute to the severity of OIs is crucial for improving patient outcomes.

Diabetes mellitus (DM) is a chronic metabolic disorder characterized by hyperglycemia resulting from defects in insulin secretion, insulin action, or both [4]. It is well established that patients with DM are at increased risk for infections due to impaired immune responses [5]. The hyperglycemic environment within these patients impairs neutrophil function, reduces chemotaxis, and alters cytokine production, leading to a decreased ability to fight infections [6]. Consequently, patients with diabetes are more susceptible to developing severe OIs and associated complications [7].

Inflammatory markers and scoring systems have been utilized to assess the severity of infections and predict clinical outcomes. The quick Sequential Organ Failure Assessment (qSOFA) score is a simplified version of the SOFA score and is used to identify patients at risk of poor outcomes due to sepsis outside the intensive care unit (ICU) [8]. The Systemic Immune–Inflammation Index (SII) combines platelet counts, neutrophil counts, and lymphocyte counts to provide a comprehensive reflection of the balance between a host’s immune and inflammatory status [9]. The derived Neutrophil-to-Lymphocyte Ratio (dNLR) is another marker of systemic inflammation and has been associated with prognosis in various infections and malignancies [10].

The evaluation of inflammatory scores such as qSOFA, SII, dNLR, and OISS in diabetic patients with odontogenic infections draws on insights from studies on diabetes in broader infectious conditions like COVID-19. Studies have established that higher SII levels are linked to an increased prevalence of diabetes (16.07%) and there is a 4% increase in the likelihood of developing diabetes per SII unit [11]. Similarly, patients with diabetes exhibit elevated qSOFA scores, highlighting their intensified response to infections [12]. Such inflammatory indices correlate with adverse outcomes, as patients with diabetes with high SII scores have shown increased mortality rates (HR 2.05 for cardiovascular deaths) [13]. These findings underscore the relevance of these scores in assessing the risk and prognosis of infections in diabetic patients, reflecting the systemic inflammatory state and its impact on infection severity [14,15].

The Odontogenic Infection Severity Score (OISS) is a clinical tool specifically designed to assess the severity of OIs [16]. It takes into account factors such as the number of affected anatomical spaces, presence of systemic symptoms, and airway compromise. Despite the availability of these scoring systems, there is limited research on their applicability in patients with diabetes with OIs.

Given the increased risk of severe infections in diabetic patients, it is essential to evaluate the effectiveness of these inflammatory scores in predicting outcomes in this population. The early identification of high-risk patients could lead to timely interventions, thereby reducing morbidity and mortality [17]. This study aims to compare the qSOFA, SII, dNLR, and OISS inflammatory scores between patients with and without diabetes with OIs and assess their predictive value for severe outcomes.

## 2. Materials and Methods

### 2.1. Study Design and Ethical Considerations

This case-control study was conducted at the “Victor Babes” University of Medicine and Pharmacy Timisoara. The study was approved by the Institutional Review Board, and all procedures were in accordance with the ethical standards of the Declaration of Helsinki. Informed consent was obtained from all participants prior to inclusion in the study.

Patient confidentiality was maintained throughout the study, and data were anonymized before analysis. The inclusion criteria were patients aged 18 years and older diagnosed with odontogenic infections requiring hospitalization and surgical intervention. Exclusion criteria included patients with immunodeficiency disorders other than diabetes, malignancies, chronic inflammatory diseases, recent trauma, or those who refused to participate.

### 2.2. Patient Selection and Grouping

A total of 123 patients diagnosed with OIs were enrolled in the study. Patients were divided into two groups based on the presence of diabetes mellitus: a diabetic group (*n* = 42) and a non-diabetic group (*n* = 81). Diabetes mellitus was confirmed based on medical history, fasting blood glucose levels, and HbA1c measurements according to the American Diabetes Association criteria [18]. Baseline demographic data, including age, gender, body mass index (BMI), smoking status, alcohol consumption, and comorbidities, were recorded. Comorbidities considered included hypertension, cardiovascular disease, chronic kidney disease, and chronic obstructive pulmonary disease. The primary site of infection, duration of symptoms, and prior antibiotic use were also documented.

### 2.3. Clinical Assessment and Scoring Systems

Upon admission, all patients underwent a comprehensive clinical examination. Vital signs, including temperature, heart rate, respiratory rate, blood pressure, and oxygen saturation, were recorded. The extent of the infection was assessed through a physical examination and imaging studies, including panoramic radiographs and computed tomography scans when necessary.

Inflammatory scores were calculated as follows: (1) qSOFA: one point each was assigned for systolic blood pressure ≤ 100 mmHg, respiratory rate ≥ 22 breaths per minute, and altered mental status (Glasgow Coma Scale < 15) [19]; (2) SII: calculated as (platelet count × neutrophil count)/lymphocyte count, using values from complete blood count (CBC) at admission [20]; (3) dNLR: calculated as neutrophil count/(white blood cell count—neutrophil count) [21]; (4) OISS: scored based on the number of fascial spaces involved, presence of systemic symptoms (fever, malaise), airway compromise, and need for ICU admission [22].

### 2.4. Laboratory Investigations

Blood samples were collected at admission for routine laboratory tests, including complete blood count (CBC), C-reactive protein (CRP), erythrocyte sedimentation rate (ESR), fasting blood glucose, HbA1c, renal function tests, and liver function tests. Neutrophil and lymphocyte counts were obtained from the CBC to calculate the SII and dNLR. Blood cultures were obtained in cases with suspected systemic infection. Glycemic control in diabetic patients was assessed using HbA1c levels. Poor glycemic control was defined as HbA1c ≥ 7% [23]. The inflammatory markers CRP and ESR were also compared between the two groups.

### 2.5. Clinical Outcomes and Statistical Analysis

Patients were followed throughout their hospital stay to monitor clinical outcomes, including duration of hospitalization, need for ICU admission, development of complications (e.g., sepsis, airway obstruction), and mortality. Surgical interventions performed included incision and drainage, tooth extractions, and debridement as necessary.

The statistical analysis was performed using SPSS version 26.0 (IBM Corp., Armonk, NY, USA). Continuous variables were expressed as means ± standard deviation (SD) and compared using the independent samples *t*-test or Mann–Whitney U test, as appropriate. Categorical variables were presented as frequencies and percentages and compared using the chi-square test or Fisher’s exact test. Receiver operating characteristic (ROC) curves were constructed to evaluate the predictive accuracy of the inflammatory scores, and the area under the curve (AUC) was calculated. Multivariate logistic regression analysis was used to identify independent predictors of severe OIs. A *p*-value < 0.05 was considered statistically significant.

## 3. Results

The mean age of the patients with diabetes was significantly higher at 58.4 ± 10.7 years compared to 45.6 ± 12.3 years for the patients who were non-diabetic (*p* < 0.001). The gender distribution was similar between the two groups, with males constituting 61.9% of the patients with diabetes patients and 60.5% of the patients who were non-diabetic (*p* = 0.872), indicating no significant gender-based differences. The mean BMI was slightly higher in the diabetic group (28.3 ± 4.5 kg/m^2^) compared to the non-diabetic group (26.8 ± 5.1 kg/m^2^), but this difference was not statistically significant (*p* = 0.084). Lifestyle factors such as smoking and alcohol use were comparable between the groups, with no significant differences being observed (*p* = 0.974 and *p* = 0.867, respectively). However, comorbidities were significantly more prevalent in the diabetic group. Hypertension was present in 47.6% of the patients with diabetes compared to 22.2% of the patients who were non-diabetic (*p* = 0.003). Similarly, cardiovascular disease was more common in the patients with diabetes (35.7% vs. 12.3%, *p* = 0.002). Chronic kidney disease was also significantly higher in the patients with diabetes (19.0% vs. 6.2%, *p* = 0.029), as presented in Table 1.

The duration of symptoms prior to admission was significantly longer in the patients with diabetes, averaging 5.8 ± 2.1 days compared to 4.3 ± 1.7 days in the patients who were non-diabetic (*p* = 0.001). This delay in presentation may be due to various factors, including decreased pain perception or delayed healthcare-seeking behavior among patients with diabetes. The mean number of fascial spaces involved was higher in the diabetic group (2.4 ± 0.9) compared to the non-diabetic group (1.7 ± 0.6), and this difference was statistically significant (*p* < 0.001).

Airway compromise, a critical concern in OIs, was observed in 23.8% of patients with diabetes, significantly higher than the 7.4% observed in the patients who were non-diabetic (*p* = 0.010). The higher incidence of airway compromise underscores the severity of infections in patients with diabetic. The OISS, which quantifies the severity of OIs, was significantly higher in patients with diabetes (mean 6.5 ± 2.8) compared to the patients who were non-diabetic (mean 4.8 ± 2.1, *p* < 0.001), as seen in Table 2.

Table 3 details the laboratory findings and inflammatory markers for both groups. The mean white blood cell (WBC) count was significantly higher in the patients with diabetes (13.5 ± 3.2 × 10^9^/L) compared to the patients who were non-diabetic (11.2 ± 2.8 × 10^9^/L, *p* < 0.001), indicating a stronger inflammatory response. Neutrophil counts were elevated in the patients with diabetes (10.4 ± 2.5 × 10^9^/L) compared to the patients who were non-diabetic (8.6 ± 2.1 × 10^9^/L, *p* < 0.001), while lymphocyte counts were lower in the patients with diabetes (1.2 ± 0.4 × 10^9^/L vs. 1.8 ± 0.5 × 10^9^/L, *p* < 0.001). Platelet counts were slightly higher in the patients with diabetes (280 ± 65 × 10^9^/L) than in the patients who were non-diabetic (250 ± 58 × 10^9^/L, *p* = 0.009).

Inflammatory markers CRP and ESR were significantly elevated in the patients with diabetes (CRP: 78.5 ± 20.7 mg/L vs. 60.3 ± 18.5 mg/L, *p* < 0.001; ESR: 52.7 ± 15.3 mm/h vs. 40.8 ± 12.9 mm/h, *p* < 0.001), suggesting a heightened systemic inflammatory response. The HbA1c levels were significantly higher in the patients with diabetes (8.2 ± 1.1%) compared to the patients who were non-diabetic (5.4 ± 0.6%, *p* < 0.001), confirming the poor glycemic control in the diabetic group.

The mean qSOFA score was significantly higher in the patients with diabetes (1.6 ± 0.7) compared to the patients who were non-diabetic (0.9 ± 0.6, *p* < 0.001), indicating a higher risk of poor outcomes. The mean SII was markedly elevated in the patients with diabetes (1423 ± 656) versus the patients who were non-diabetic (1036± 482, *p* < 0.001). The higher SII in the patients with diabetes reflects a pronounced systemic inflammatory and immune response imbalance. Similarly, the dNLR was significantly higher in the patients with diabetes (5.9 ± 1.8) compared to the patients who were non-diabetic (3.6 ± 1.2, *p* < 0.001). An elevated dNLR is associated with a pro-inflammatory state and has been linked to a poor prognosis in infections (Table 4).

**Figure 1:**
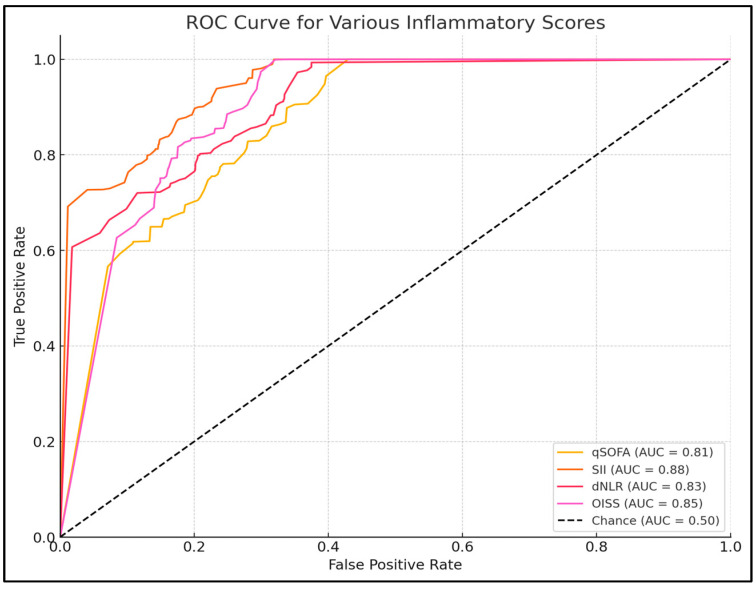
ROC curve analysis for COVID-19 inflammatory scores.

The SII demonstrated the highest predictive accuracy with an AUC of 0.88 (95% CI: 0.80–0.95), indicating excellent discriminative ability. An optimal cutoff value of 1560 yielded a sensitivity of 85.7% and specificity of 80.2%. The qSOFA score had an AUC of 0.81, with an optimal cutoff of 1.5, achieving a sensitivity of 78.6% and specificity of 75.3%. The dNLR showed an AUC of 0.83, with a cutoff of 5.0, sensitivity of 80.9%, and specificity of 77.8%. The OISS had an AUC of 0.85, indicating good predictive accuracy, with an optimal cutoff of 9.0 (Table 5 and Figure 1).

Table 6 illustrates the clinical outcomes observed in both groups. ICU admission was significantly more frequent among diabetic patients (28.6%) compared to non-diabetic patients (9.9%, *p* = 0.007). The development of sepsis was also higher in patients with diabetes (23.8% vs. 7.4%, *p* = 0.010). The mean length of hospital stay was significantly longer for the patients with diabetes, averaging 9.5 ± 2.8 days compared to 6.3 ± 2.1 days for patients who were non-diabetic (*p* < 0.001). Mortality was higher in the diabetic group (7.1%) compared to the non-diabetic group (1.2%), although this difference did not reach statistical significance (*p* = 0.086), possibly due to the small number of deaths (Table 6).

## 4. Discussion

### 4.1. Analysis of Findings

This study demonstrates that patients with diabetes with odontogenic infections present with more severe disease and worse clinical outcomes compared to patients who are non-diabetic. The significantly higher OISS scores in the patients with diabetes indicate more extensive infections, increased fascial space involvement, and a greater risk of airway compromise. These findings are consistent with previous studies that have shown diabetes mellitus as a risk factor for severe OIs [24].

Inflammatory markers and scores were significantly elevated in the patients with diabetes. The higher WBC counts, CRP, ESR, and particularly the elevated SII and dNLR, reflect a heightened inflammatory state in patients with diabetes. The SII, which incorporates neutrophil, lymphocyte, and platelet counts, showed the highest predictive accuracy for severe outcomes, with an AUC of 0.88. This suggests that SII is a valuable tool for assessing the severity of OIs in patients with diabetes. The qSOFA and dNLR also demonstrated good predictive value, reinforcing their utility in clinical practice. The qSOFA score is a quick and simple tool that can be used at the bedside to identify patients at risk of poor outcomes.

Both studies by Neal et al. [16] and Haghighatpanah et al. [23] emphasize the multifaceted utility of the Odontogenic Infection Severity Score (OISS). Their research found that patients with an OISS ≥ 5 not only faced nearly four times the likelihood of experiencing difficult intubations (Odds Ratio 3.70, 95% CI 1.19–11.45) but also incurred significantly higher hospital costs. This correlation highlights the clinical importance of OISS in preoperative planning, where a sensitivity of 69% and a specificity of 63% suggest a moderate discriminative power for anticipating intubation challenges (*p* = 0.018). Simultaneously, the economic study underscores the financial impact, with high OISS linked to increased treatment costs and identified predictors such as male sex, elevated blood glucose, and high WBC counts further aligning with severe infection markers. These findings advocate for the integration of OISS in clinical workflows to enhance both patient care and resource management.

In the studies by Bond et al. [25] and Kusumoto et al. [26], detailed numerical data illustrate the clinical utility of assessing odontogenic infections (OIs). Bond et al. reported that patients with deep OIs had a significantly increased risk of sepsis, with a relative risk of 5.4 times greater than those with superficial infections (95% CI: 1.51 to 19.27, *p* = 0.004) and an odds ratio of 9.13 for a qSOFA score >0, indicating a strong predictor of sepsis risk (95% CI: 2.48 to 33.55, adjusted *p* < 0.001). Similarly, Kusumoto et al. found that the Laboratory Risk Indicator for Necrotizing Fasciitis score, with a cutoff of 6, and a CRP + neutrophil-to-lymphocyte ratio with a cutoff of 27, were effective for diagnosing severe cases, and their decision tree analysis, using a systemic immune-inflammation index (SII) cutoff of ≥282, achieved a diagnostic accuracy of 89.3%. These findings emphasize the importance of specific clinical and laboratory markers in stratifying the risk and aiding in the early detection of potentially severe outcomes in patients with OIs.

In their retrospective studies, Hammad et al. [27] found that, while C-reactive protein (CRP) levels and body temperature at admission did not significantly differ between groups with varying severity of OIs, the white blood cell (WBC) count and glucose levels were significantly higher in patients with more severe infections (*p* = 0.001 and *p* = 0.036, respectively). This suggests that WBC and glucose are more reliable indicators of severe odontogenic infections. In a similar manner, Pricop et al. [28] demonstrated the utility of the Systemic Immune–Inflammation Index (SII) and Symptom Severity score (SS) in predicting severe outcomes such as sepsis and Systemic Inflammatory Response Syndrome (SIRS) in OI patients. They reported that higher SII and SS values correlated significantly with an increased risk of sepsis and SIRS, supported by a statistically significant positive correlation (r = 0.6314) and distinct differences in mean values between their severity groups (SII: 696.3 in less severe vs. 2312.4 in more severe; SS: 6.1 vs. 13.6). Both studies underscore the importance of specific clinical and laboratory parameters in assessing the risk and managing the treatment of severe odontogenic infections effectively.

In their retrospective studies, Gallagher et al. [29] and Rosca et al. [30] explored the prognostic value of biomarkers in predicting the severity and outcomes of odontogenic infections (OIs). Gallagher et al. [29] focused on the Neutrophil to Lymphocyte Ratio (NLR) as a marker for deep neck space infections secondary to OIs, finding that a higher NLR was significantly associated with an increased length of stay (LOS). Specifically, they reported that an NLR ≥ 4.65 predicted an LOS ≥ 2 days, with an area under the curve (AUC) of 0.639 for this threshold, underscoring its moderate predictive validity (*p* ≤ 0.01). Similarly, Rosca et al. [30] investigated the combined C-reactive protein and Neutrophil-to-Lymphocyte Ratio (CRP-NLR) to evaluate infection severity. They found that higher CRP-NLR scores were significantly associated with severe outcomes, such as an increased incidence of sepsis in more severely infected patients (22.2% vs. 7.4%, *p*-value = 0.030). Their ROC analysis yielded a high AUC of 0.889, indicating an excellent sensitivity (79.6%) and specificity (85.1%) in predicting severe odontogenic infections. Both studies illustrate the clinical utility of NLR and CRP-NLR as effective biomarkers for assessing the risk and guiding the management of patients with severe OIs, suggesting these markers could enhance decision-making in dental and maxillofacial interventions.

The clinical utility of these findings lies in their potential to enhance the management of odontogenic infections, particularly in the patients with diabetes who are at increased risk for severe complications. By incorporating inflammatory scores such as qSOFA, SII, dNLR, and OISS into routine clinical assessments, healthcare providers can identify high-risk patients more effectively. This early identification can facilitate timely interventions, such as aggressive antibiotic therapy and surgical management, possibly preventing the progression to more severe outcomes like sepsis or ICU admission. Furthermore, these scores could assist in stratifying patients based on risk, allowing for more personalized care plans and resource allocation in hospital settings, thereby improving patient outcomes and optimizing healthcare resource utilization.

### 4.2. Study Limitations

While this study provides valuable insights into the predictive value of inflammatory scores in patients with and without diabetes with odontogenic infections, several limitations must be acknowledged. First, the sample size, particularly in the diabetic group, may not be sufficiently large to generalize the findings across diverse populations. Second, the study design does not account for potential confounders such as variations in treatment protocols across patients, which could influence the outcomes. Third, the reliance on hospital-based data might introduce selection bias, as it excludes milder cases managed in outpatient settings. Additionally, the observational nature of the study limits the ability to establish causality between inflammatory scores and clinical outcomes. Finally, the study’s setting in a single institution may not reflect the broader clinical practice, potentially limiting the applicability of the results to other healthcare settings.

## 5. Conclusions

In conclusion, this study demonstrates that inflammatory scores such as qSOFA, SII, dNLR, and OISS may provide valuable predictive information regarding the severity of outcomes in patients with and without diabetes with odontogenic infections. In particular, higher scores were consistently associated with more severe outcomes in patients with diabetes, underscoring the need for early and aggressive management strategies in this vulnerable population. While these findings suggest that inflammatory scores can aid in the early identification of high-risk patients, thereby potentially reducing morbidity and mortality, further research with larger, multicentric cohorts is necessary to validate these scores and refine their predictive accuracy across different clinical settings and populations.

## Figures and Tables

**Table 1:** Demographic and clinical characteristics of patients with and without diabetes with odontogenic infections.

Variables	Patients with Diabetes (*n* = 42)	Controls (*n* = 81)	*p*-Value
Age (years, mean ± SD)	58.4 ± 10.7	45.6 ± 12.3	<0.001 *
Gender (Male, *n* (%))	26 (61.9%)	49 (60.5%)	0.872
BMI (kg/m^2^, mean ± SD)	28.3 ± 4.5	26.8 ± 5.1	0.084
Smoking (*n* (%))	18 (42.9%)	35 (43.2%)	0.974
Alcohol Use (*n* (%))	12 (28.6%)	22 (27.2%)	0.867
Hypertension (*n* (%))	20 (47.6%)	18 (22.2%)	0.003 *
Cardiovascular Disease (*n* (%))	15 (35.7%)	10 (12.3%)	0.002 *
Chronic Kidney Disease (*n* (%))	8 (19.0%)	5 (6.2%)	0.029 *

BMI—body mass index; SD—standard deviation; *—statistically significant.

**Table 2:** Clinical presentation and Odontogenic Infection Severity Score (OISS).

Variables	Diabetic Patients (*n* = 42)	Controls (*n* = 81)	*p*-Value
Duration of Symptoms (days, mean ± SD)	5.8 ± 2.1	4.3 ± 1.7	0.001 *
Number of Fascial Spaces Involved (mean ± SD)	2.4 ± 0.9	1.7 ± 0.6	<0.001 *
Airway Compromise (*n* (%))	10 (23.8%)	6 (7.4%)	0.01 *
OISS (mean ± SD)	6.5 ± 2.8	4.8 ± 2.1	<0.001 *

SD—standard deviation; OISS—Odontogenic Infection Severity Score; *—statistically significant.

**Table 3:** Laboratory findings and inflammatory markers in patients with and without diabetes.

Variables	Patients with Diabetes (*n* = 42)	Controls (*n* = 81)	*p*-Value
WBC (×10^9^/L, mean ± SD)	13.5 ± 3.2	11.2 ± 2.8	<0.001 *
Neutrophil Count (×10^9^/L)	10.4 ± 2.5	8.6 ± 2.1	<0.001 *
Lymphocyte Count (×10^9^/L)	1.2 ± 0.4	1.8 ± 0.5	<0.001 *
Platelet Count (×10^9^/L)	280 ± 65	250 ± 58	0.009 *
CRP (mg/L, mean ± SD)	78.5 ± 20.7	60.3 ± 18.5	<0.001 *
ESR (mm/h, mean ± SD)	52.7 ± 15.3	40.8 ± 12.9	<0.001 *
HbA1c (%, mean ± SD)	8.2 ± 1.1	5.4 ± 0.6	<0.001 *

SD—standard deviation; *—statistically significant.

**Table 4:** Inflammatory scores at admission in patients with and without diabetes.

Scores	Patients with Diabetes (*n* = 42)	Controls (*n* = 81)	*p*-Value
qSOFA Score (mean ± SD)	1.6 ± 0.7	0.9 ± 0.6	<0.001 *
SII (mean ± SD)	1423 ± 656	1036 ± 482	<0.001 *
dNLR (mean ± SD)	5.9 ± 1.8	3.6 ± 1.2	<0.001 *

SD—standard deviation; *—statistically significant.

**Table 5:** Receiver operating characteristic (ROC) curve analysis for inflammatory scores predicting severe outcomes.

Score	AUC	Optimal Cutoff	Sensitivity (%)	Specificity (%)	*p*-Value
qSOFA	0.81	1.5	78.6	75.3	<0.001 *
SII	0.88	1560	85.7	80.2	<0.001 *
dNLR	0.83	5	80.9	77.8	<0.001 *
OISS	0.85	9	82.5	79.0	<0.001 *

AUC—area under curve; *—statistically significant.

**Table 6:** Clinical outcomes in patients with and without diabetes.

Outcomes	Patients with Diabetes (*n* = 42)	Controls (*n* = 81)	*p*-Value
ICU Admission (*n* (%))	12 (28.6%)	8 (9.9%)	0.007 *
Sepsis Development (*n* (%))	10 (23.8%)	6 (7.4%)	0.01 *
Length of Hospital Stay (days, mean ± SD)	9.5 ± 2.8	6.3 ± 2.1	<0.001 *
Mortality (*n* (%))	3 (7.1%)	1 (1.2%)	0.086

ICU—intensive care unit; *—statistically significant.

## Data Availability

The data presented in this study are available on request from the corresponding author.
